# Navigating Crown-Root Fracture Complexities in an Adolescent

**DOI:** 10.7759/cureus.71406

**Published:** 2024-10-13

**Authors:** Nishanthi Selvaraj, Noraida Mamat, Haslina Taib

**Affiliations:** 1 Unit of Pediatric Dentistry, School of Dental Sciences, Universiti Sains Malaysia, Kota Bharu, MYS; 2 Unit of Periodontics, School of Dental Sciences, Universiti Sains Malaysia, Kota Bharu, MYS

**Keywords:** crown-root fracture, endodontic treatment, gingivectomy, pediatric dental trauma, supracestal tissue attachment

## Abstract

Complicated crown-root fracture is a severe dental injury that affects the enamel, dentine, cementum, and pulp, representing a small percentage of dental trauma cases in permanent teeth. This case report discusses the management of a complicated crown-root fracture to the maxillary left central incisor in a 14-year-old male patient following a motor vehicle accident. The fracture extended subgingivally, complicating access and restoration. Initial treatment attempts by a general dentist were unsuccessful due to the repeated dislodgement of restorations. A multidisciplinary approach was taken and options such as orthodontic extrusion, gingivectomy, and surgical extrusion were considered. Root canal treatment followed by gingivectomy was performed to expose the fracture line for restoration. While the fracture line for restoration was successfully exposed, the intrinsic discoloration was overlooked. The key to treatment success was the consideration of supracrestal tissue attachment, a crucial factor in maintaining periodontal health. Regular follow-up is essential to monitor the tooth’s endodontic-periodontic status and ensure lasting success.

## Introduction

A complicated crown-root fracture is a severe dental injury defined as a fracture involving enamel, dentine, cementum, and pulp [1]. This type of injury is particularly significant due to its complexity and the extensive damage it causes to the tooth structure. Such fractures account for approximately 5% of all dental trauma incidents in permanent dentition, highlighting their relative rarity but significant impact [2].

Crown-root fractures in anterior teeth are typically the result of direct trauma [3]. This could occur from various incidents such as sports injuries, falls, or accidents [4]. The nature of the fracture often extends below the gingival margin or even subcrestally, making it more challenging to manage due to the inaccessibility and the involvement of vital tooth structures. Communication with the oral cavity to the pulp and periodontal ligament permits bacterial invasion. This continuous bacterial invasion triggers inflammation in both the pulp and periodontal ligament, which can impede the natural healing process of these crown-root fractures [5].

The complexity of a crown-root fracture means that treatment approaches can vary significantly, depending on factors such as the effect of the fracture on supracrestal tissue attachment, stage of root development, stage of tooth eruption, pulpal status, the degree of adaptation of the fragment to the remaining tooth, and concomitant alveolar bone injury [6,7]. The complicated crown-root fracture management is challenging and controversial, especially in children. Treatment options must balance preserving the tooth and its supporting structures with the long-term growth and development of the child's dentition. This case report aims to illustrate the complexities of managing a complicated crown-root fracture in a young patient.

## Case presentation

A healthy 14-year-old Malay boy was referred to the Paediatric Dentistry Unit for the completion of root canal treatment and permanent restoration of the maxillary central incisor. Two months earlier, he had been involved in a motor vehicle accident, resulting in a complicated crown-root fracture of the affected tooth. Initially, he was treated by a general dentist for pulp extirpation of the tooth. The dentist attempted to place a permanent restoration before completing the root canal treatment to achieve a good coronal seal. However, several episodes of restoration dislodgement were reported.

**Figure 1:**
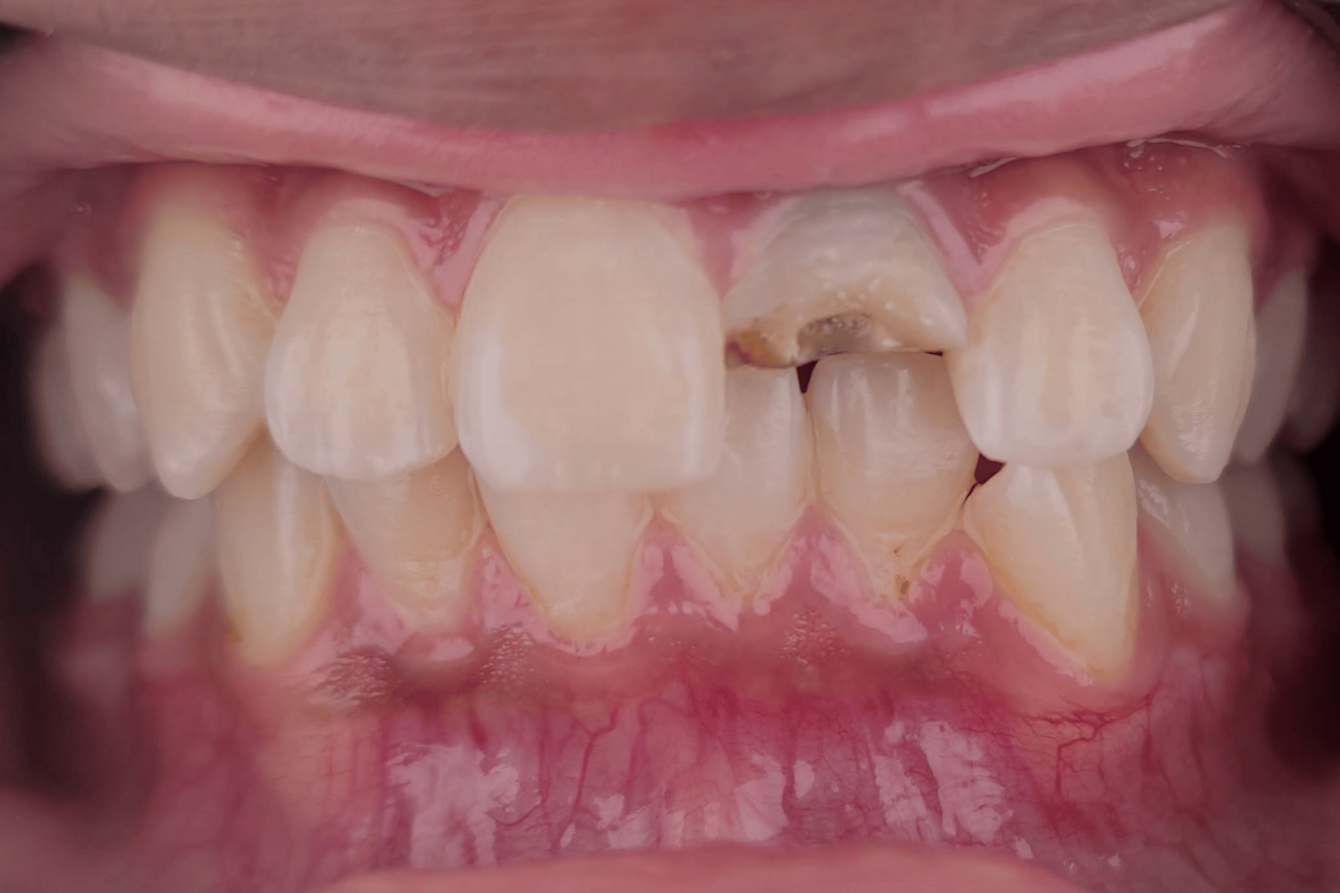
Frontal view showing fracture of the maxillary left central incisor

**Figure 2:**
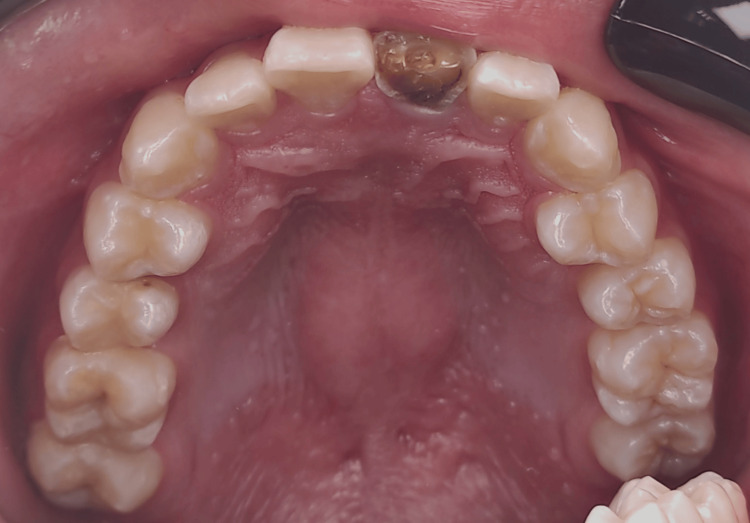
Upper occlusal view; Fracture extending subgingivally on the palatal maxillary left central incisor

**Figure 3:**
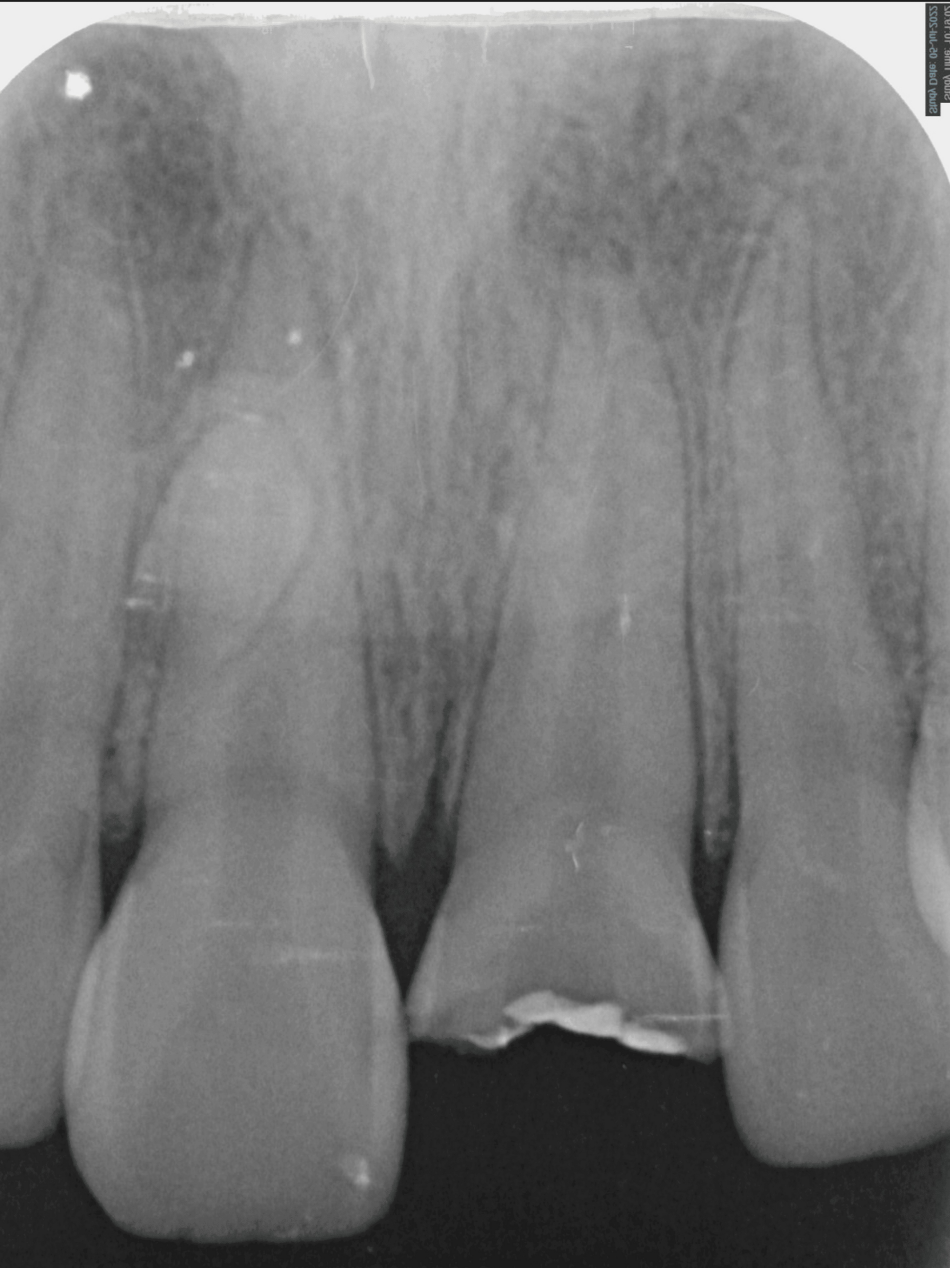
Periapical radiograph showing the fractured maxillary left central incisor

**Figure 4:**
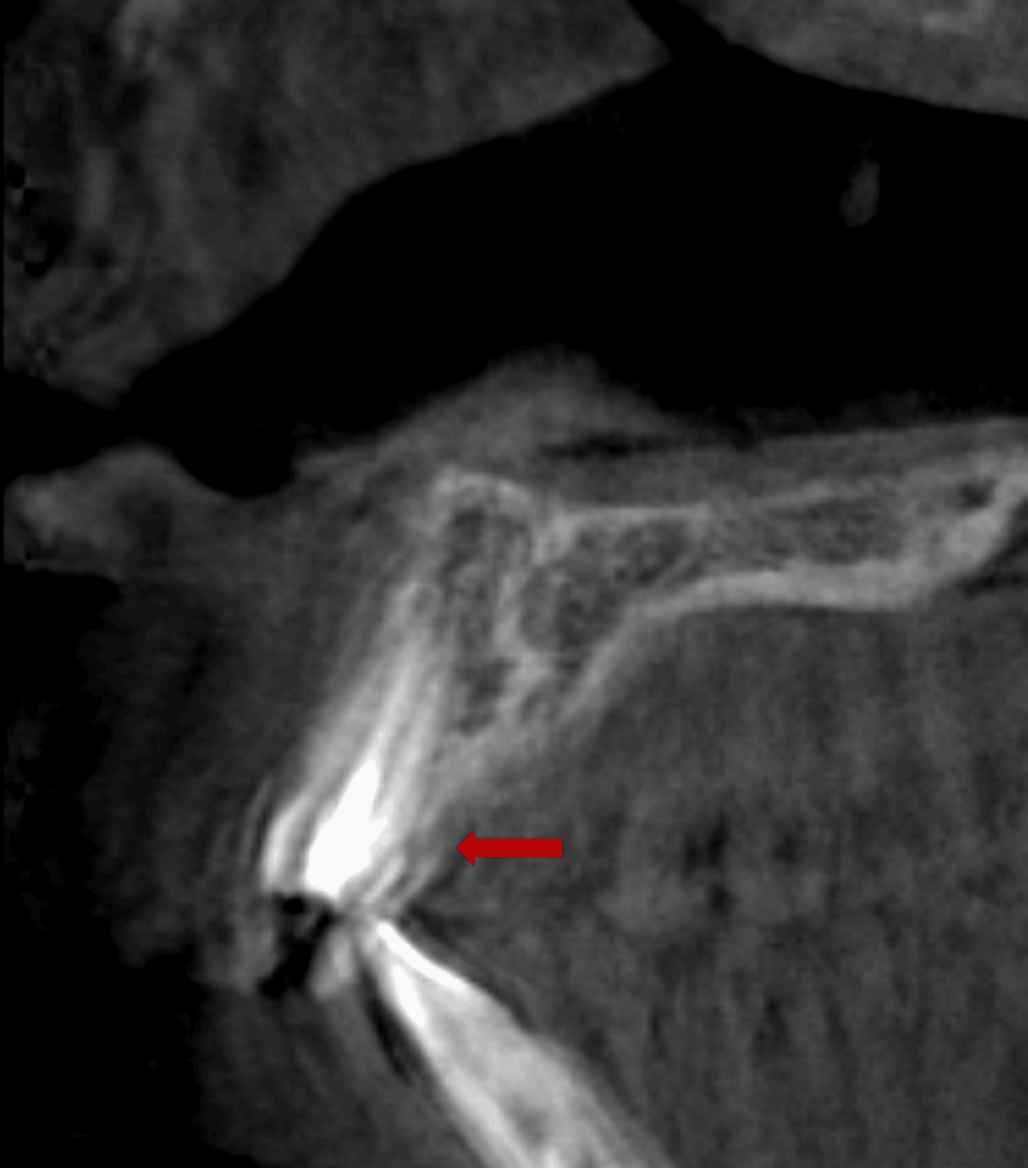
Cone-beam CT (sagittal view) showing the fracture line with overhanging remaining temporary restoration

The intra-oral examination revealed a fractured maxillary left central incisor, affecting half of the crown and exposing the pulp orifice (Figure 1). No mobility or abscess were noted. The fracture line extended subgingivally on the palatal surface (Figure 2), and the tooth appeared discoloured. Upon investigation, the periapical radiograph showed closed apex and an intact periodontal ligament space with mild periapical radiolucency. However, no visible extension of the fracture was noted (Figure 3). Further examination with cone-beam computed tomography (CBCT) revealed the extent of the fracture line, approximately 3 mm from the cementoenamel junction (CEJ), and the overhanging of the remaining previous restoration was also noted (Figure 4).

After a comprehensive discussion with the orthodontist and periodontist, several treatment options were presented to address the case. The options included orthodontic extrusion, gingivectomy or crown lengthening, and surgical extrusion. Each option was carefully evaluated in terms of risks, benefits, and associated costs. After considering these options, the patient and their parents opted for a gingivectomy followed by a crown build-up. This choice was made with the aim of achieving a functional and aesthetic outcome while balancing the associated risks and costs.

**Figure 5:**
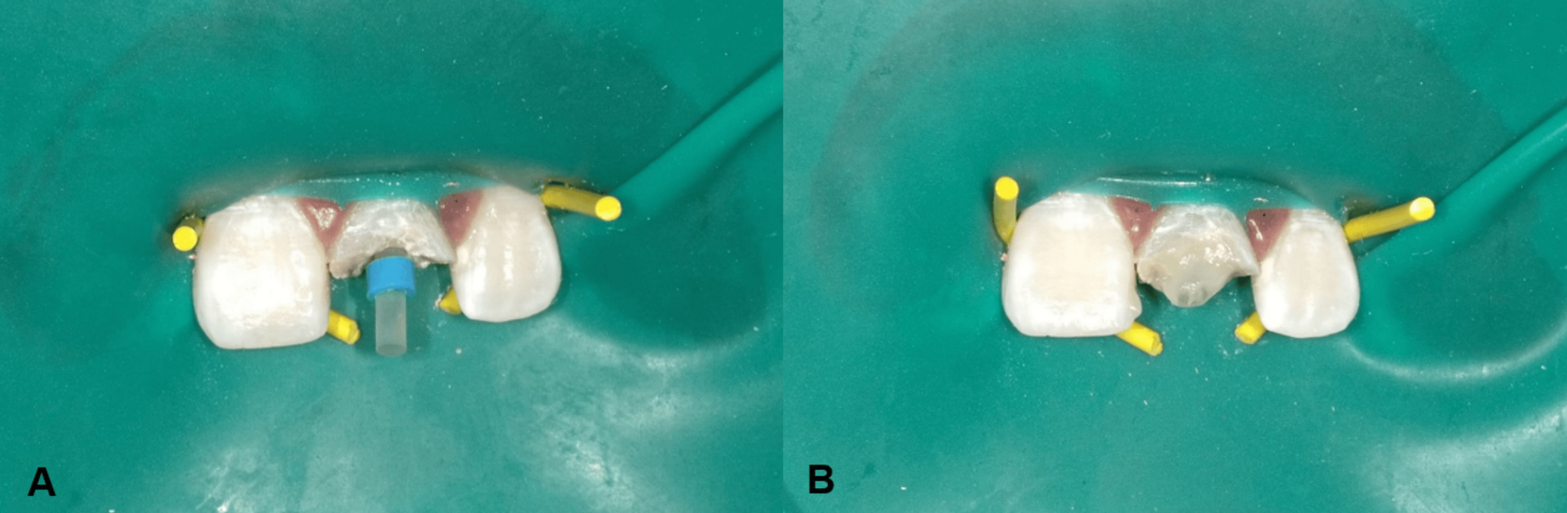
(A) Try-in of pre-fabricated post. (B) Cementation of pre-fabricated post

**Figure 6:**
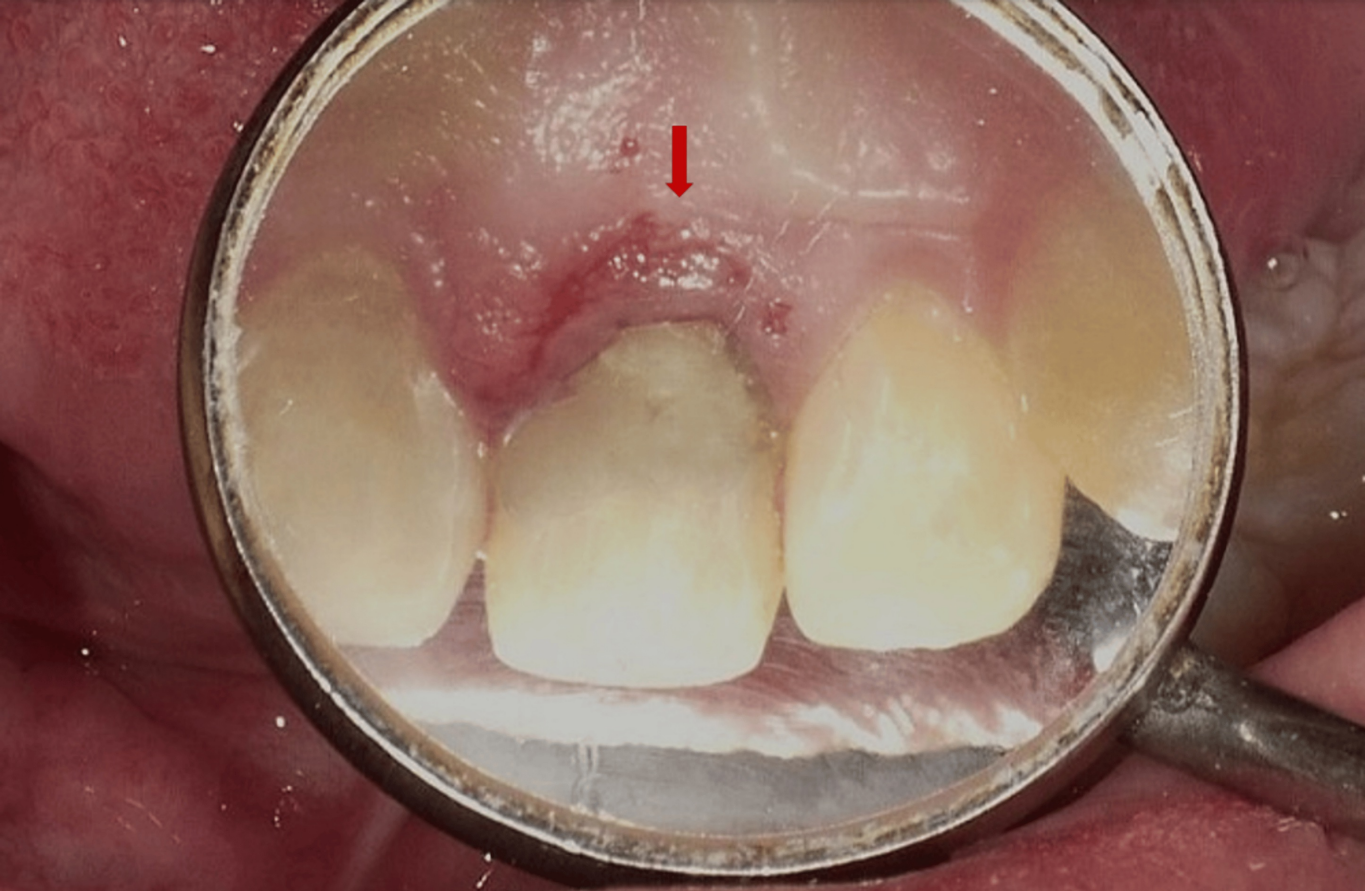
Arrow shows pocket marking

**Figure 7:**
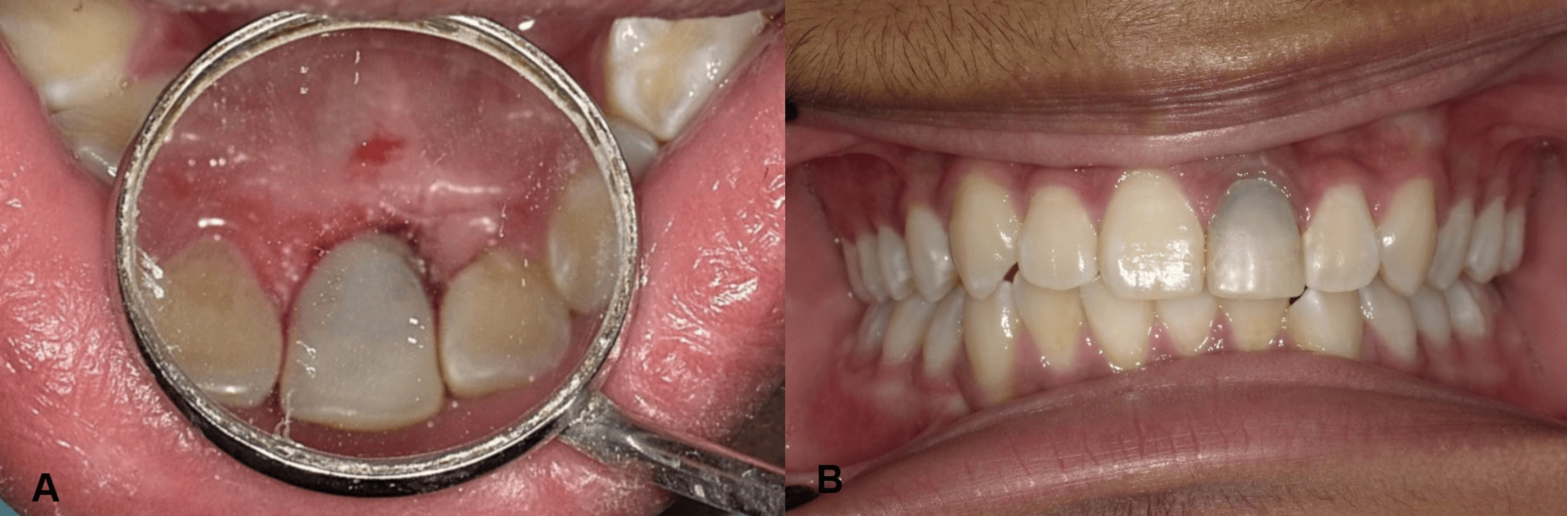
(A) Composite restoration on the palatal surface. (B) Composite restoration on frontal view

Treatment of the maxillary left central incisor began with root canal therapy to address any underlying infection and prepare the tooth for further restoration. A pre-fabricated fiber post was placed during a subsequent visit to provide structural support (Figure 5), and a temporary restoration was done to protect the tooth. The gingivectomy procedure was performed under local anesthesia at the following appointment. Prior to the procedure, bone sounding was carried out to determine the bone level on the palatal side of the affected tooth, indicating that no bone removal or ostectomy was required. The amount of supracrestal gingival tissue to be excised was marked by creating bleeding spots (Figure 6). A gingivectomy was then performed using a #15 scalpel blade to remove the necessary amount of supracrestal gingival tissue, thereby enhancing access to the tooth structure. Haemostasis was achieved by compressing wet gauze on the surgical area for a few minutes. After removing the overhanging temporary restoration, the fracture line was identified. Finally, on the same day, a composite restoration was completed to restore the tooth's function (Figure 7).

**Figure 8:**
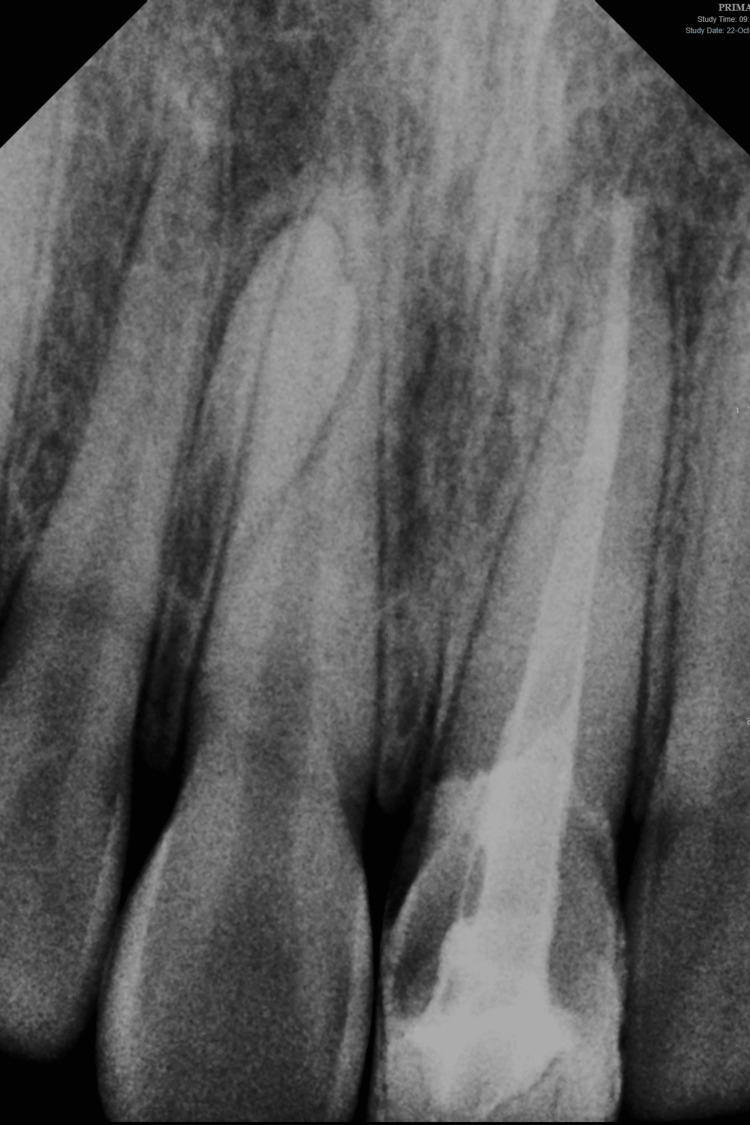
Periapical radiograph at one-month review

**Figure 9:**
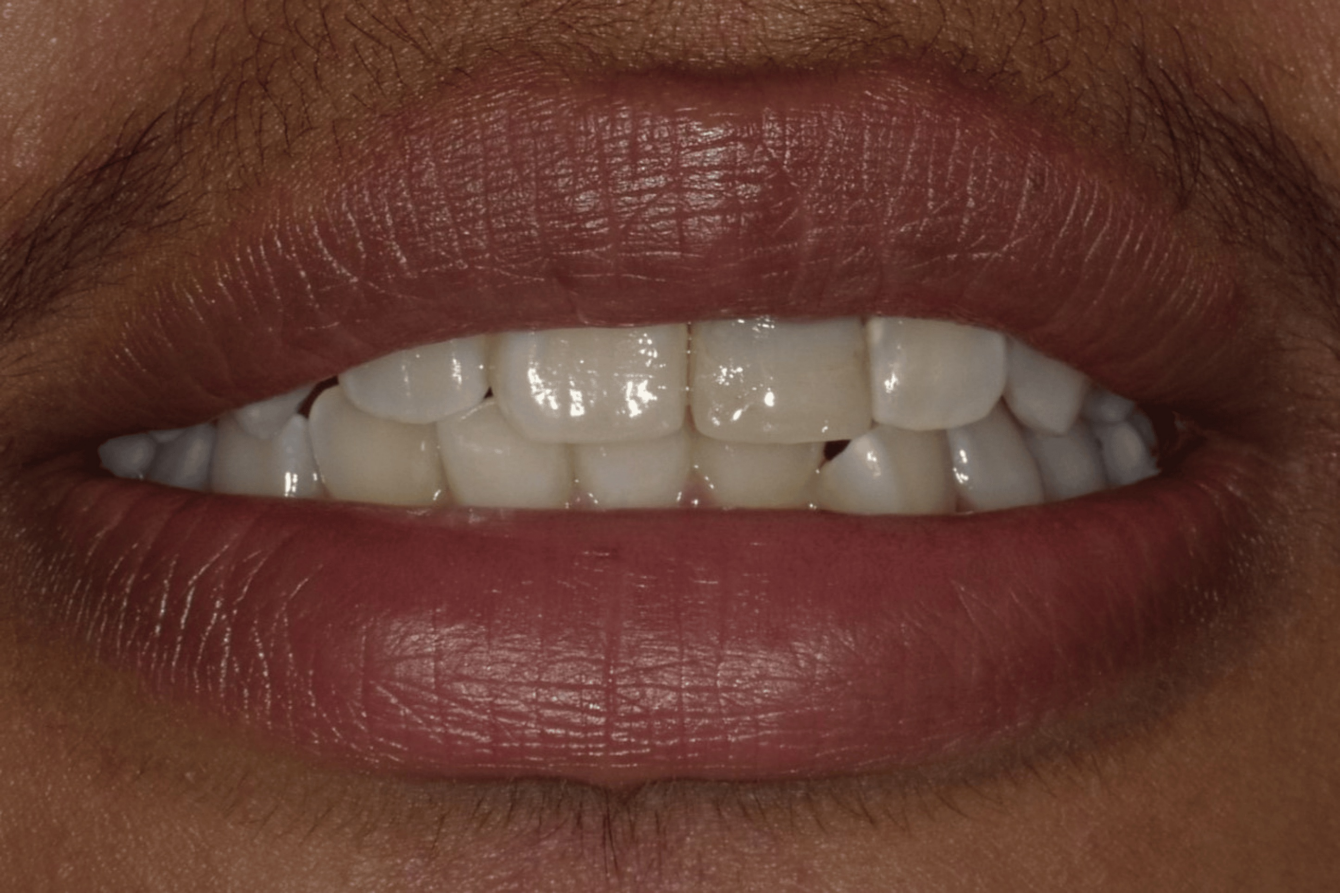
At the low smile line, the discoloration is not visible

At the one-year follow-up appointment, healing was uneventful, and the patient exhibited no signs or symptoms of complications, either clinically or radiographically (Figure 8). Although the discoloration of the treated tooth had not been fully addressed, the patient expressed satisfaction with the overall appearance of their smile (Figure 9). It was discussed that, to more effectively address the discoloration, a future restoration with an opaque crown could be considered to mask the discoloration and further enhance the aesthetic outcome.

## Discussion

Restoring crown-root fractured incisors in children presents a formidable challenge for clinicians, as it involves addressing functional, aesthetic, and biological concerns simultaneously [8,9]. The treatment strategy, which consists of a combination of endodontic, periodontal, and restorative procedures, is determined by factors like pulp exposure and involves the supporting periodontal structures. Invasion of the supracrestal tissue attachment by the fracture poses a clinical challenge for restoration [10]. Thus, in cases of complicated crown-root fractures, endodontic treatment should be carried out before addressing any periodontal procedures [9].

Surgical crown lengthening or gingivectomy are viable treatment options for exposing a subgingival fracture on a tooth, making it possible to restore the tooth effectively [11]. These procedures focus on removing or reshaping the gingival tissue, and in some cases, the underlying bone, to ensure that the fracture line is visible and accessible for treatment. Orthodontic extrusion and intentional replantation are the other treatment options available for managing such cases [1,12].

In this case, where the fracture line extends subgingivally on the palatal surface, gingivectomy emerges as an ideal treatment option to convert the subgingival fracture to a supragingival one [2]. This procedure allows for the establishment of a proper finish margin for the restoration [9,13]. Several key reasons led to the selection of this treatment option. Firstly, since the fracture is located on the palatal side, the procedure does not involve the aesthetic area of the tooth. Gingivectomy is also advantageous due to its relatively short treatment time, allowing the fracture to be exposed and treated promptly, which is crucial for preventing further complications such as failure of endodontic treatment.

Moreover, it is a cost-effective solution, offering a more practical treatment alternative to orthodontic extrusion. Gingivectomy typically involves the removal of gingival tissue below the fracture line without altering the underlying bone. This leads to a quicker healing process and reduces post-operative discomfort for the patient [14]. These factors collectively make gingivectomy a practical and efficient approach for managing subgingival fractures on the palatal surface, balancing effectiveness, cost, and patient comfort.

When considering gingivectomy as a treatment option, the supracrestal tissue attachment and the available sound bone are two critical factors to assess. Supracrestal tissue attachment was previously known as biological width and refers to the junctional epithelium and supracrestal connective tissue [15]. It protects against infection and other foreign materials, acting dynamically to maintain a constant distance. Violation of the supracrestal soft tissue may result in apical migration of junctional epithelium leading to localized crestal bone loss, gingival recession, pocket formation, and localized gingival hyperplasia [16,17]. Gingivectomy is recommended when the final restoration is positioned at least 3 mm above the alveolar bone crest [17]. Therefore, a proper examination and treatment plan in collaboration with periodontists is essential to avoid such complications.

However, in this case, the management of discoloration was not fully addressed. This intrinsic discoloration may be due to pulp necrosis, remnants of pulp tissue following endodontic treatment, root canal irrigants, and sealers [18]. Prosthodontic options such as ceramic veneers or crowns can be considered for future treatment.

## Conclusions

Monitoring the tooth's endodontic-periodontic status over time is essential for a favorable prognosis. Regular follow-up visits should include clinical examinations and radiographs to check for signs of healing or complications, such as persistent infection, root resorption, or periodontal disease. Any issues identified during follow-up can be addressed promptly to maintain the health and function of the tooth. Combining the expertise of various dental specialists and employing a comprehensive treatment approach can significantly improve the prognosis for these challenging cases.
